# Habituation, sensitization, and Pavlovian conditioning

**DOI:** 10.3389/fnint.2014.00013

**Published:** 2014-02-11

**Authors:** Münire Özlem Çevik

**Affiliations:** Psikoloji Bolumu, TOBB Ekonomi ve Teknoloji UniversitesiAnkara, Turkey

**Keywords:** proboscis extension response, habituation, *Drosophila melanogaster*, olfactory conditioning, mushroom bodies, dopamine

## Abstract

In this brief review, I argue that the impact of a stimulus on behavioral control increase as the distance of the stimulus to the body decreases. Habituation, i.e., decrement in response intensity repetition of the triggering stimulus, is the default state for sensory processing, and the likelihood of habituation is higher for distal stimuli. Sensitization, i.e., increment in response intensity upon stimulus repetition, occurs in a state dependent manner for proximal stimuli that make direct contact with the body. In Pavlovian conditioning paradigms, the unconditioned stimulus (US) is always a more proximal stimulus than the conditioned stimulus (CS). The mechanisms of associative and non-associative learning are not independent. CS−US pairings lead to formation of associations if sensitizing modulation from a proximal US prevents the habituation for a distal anticipatory CS.

Identification of the necessary and sufficient conditions for the formation of associations has been a driving influence on learning theory and research. In Pavlovian conditioning, a conditioned stimulus (CS) acquires the ability to trigger a new response by virtue of being paired with an unconditioned stimulus (US), which by definition is biologically important and capable of triggering an innate reflex. Starting with British associationism, early theories of conditioning were based on the premise that temporal contiguity was both necessary and sufficient for stimulus associations (Gormezano and Kehoe, 1981). Although the temporal coincidence of the CS−US pair is still accepted to be necessary, research since late 1960's presented irrevocable challenges to its sufficiency for the formation of associations (Durlach, 1989). Of particular importance was the discovery of blocking (Kamin, 1968), where an association fails to be formed in spite of the seamless temporal contiguity between the CS and the US, if the CS is presented in a compound with another CS that had previously been associated with the same US. Blocking had a major bearing on the development of the contingency theory of associative learning (Rescorla and Wagner, 1972), which has been a major breakthrough that diverted the focus of learning research from physical properties (e.g., intensity and temporal coincidence) to the signal value [e.g., predictiveness, informativeness, (un)expectedness] of the to-be-associated stimuli (Rescorla and Holland, 1988; Gallistel and Matzel, 2013).

The signal value of a stimulus is correlated with its potential to support responsiveness, which for a given set of physical parameters depends largely on the history of non-associative learning, i.e., habituation and sensitization for the stimulus. Habituation refers to the reduction in the probability or amplitude of responding that is observed upon inconsequential stimulus repetition. For example, repeated delivery of an odor at constant inter-stimulus-intervals (ISI) would eventually lead to the habituation of the response that is initially triggered by the odor. I say eventually, because depending on the parameters of the stimulation protocol (e.g., odor concentration, frequency of odor presentation), a temporary increment in responsiveness might initially be observed. If, however, an appetitive gustatory stimulus (e.g., sugar) is repeated with the same ISI, depending again on the concentration of sugar, frequency of stimulation, and the physiological state of the organism, this protocol is likely to result in an increment in the probability of responding, i.e., sensitization. Finally if odor and sugar are paired instead of being presented separately, the standard paradigm for Pavlovian conditioning would ensue, where the two stimuli would now be termed conditioned (CS) and unconditioned stimuli (US), respectively. Hence, associative learning can be suggested to entail an evasion from habituation for the CS as it signals the arrival of a sensitizing event, the US, and a conditioned response (CR) would then be triggered during the CS in anticipation of the US.

It follows that whether or not coincident pairings of the same CS and US will yield associative learning is influenced by the signal value, or equivalently, on the history of habituation and sensitization prior to conditioning. If, for example, a CS is repeatedly presented to yield habituation prior to conditioning, then its signal value would be reduced, which would in turn reduce subsequent associative learning relative to a control condition where the CS is presented *de novo* during Pavlovian pairings. Indeed, the strength of associations decreases if organisms are pre-exposed to the CS before conditioning (Reiss and Wagner, 1972; Hall, 2001). Similarly, efficacy of a US can be potentiated if conditioning is preceded by a sensitizing treatment (LoLordo and Randich, 1981), and vice versa (see Pearce and Hall, 1980; Franklin and Hall, 2011, for explanations based on context-conditioning during US preexposure). In the same vein, the failure of a US to support new associations under a blocking paradigm can be correlated with the reduction in sensitization (or the “surprise value” of the Rescorla-Wagner model) evoked by a signaled (as opposed to an unexpected) US, and indeed, US efficacy is known to decrease with extended associative training (Rescorla and Wagner, 1972).

The suggestion that conditioning emerges as a sensitizing stimulus (US) exerts a modulatory influence to prevent the habituation for another stimulus (CS) begs the identification of stimulus characteristics that determine whether habituation or sensitization will occur upon repetition. So, are there any independent criteria for predicting *a priori* if a stimulus can incite sensitization or habituation, or equivalently, are there any inherent properties that assign a CS− or US-like function to a stimulus?

## The impact of a stimulus decreases with its distance from the body

Only a small number of model paradigms for Pavlovian conditioning endured the test of time in vertebrate and invertebrate species. The limiting factor is the scarcity of stimuli that can function as a US. In all paradigms that yield reliable conditioning, the US is a proximal stimulus that comes into direct contact with the body. For example, in appetitive conditioning paradigms, food is the most frequently used US which supports conditioned approach or discrimination via its gustatory or nutritive properties. In aversive conditioning, the US is most often a pain inflicting stimulus (e.g., heat or electric shock) that is delivered via the tactile domain. In contrast, the CS's consist of distal inputs from the visual, auditory, or olfactory domains. Distal stimuli are conspicuously ineffective as US's even after they have acquired the potential to support conditioned responding through higher-order conditioning (Rescorla, 1973). In fact, there does not exist a robust model paradigm which utilizes a distal stimulus as the US, and a proximal stimulus as the CS. The more proximal nature of the US relative to the CS is true even for paradigms where the CS and the US are first encountered through the same sensory modality. For example, in taste preference conditioning, the gustatory stimulus is the CS, and the nutritive value of food which requires further digestive processing, is the US (de Araujo, 2011; Fujita and Tanimura, 2011). Similarly, digestion-related malaise functions as the US in conditioned taste aversion (Garcia and Koelling, 1966).

The distinction between distal and proximal stimuli is non-trivial because it matches the temporal order of events as the animal moves and/or the environment changes. For example, one can smell an apple before/without tasting it, but not vice versa. In general, biologically important objects (e.g., food, predators, aggressive opponents, potential mates, sharp objects) are detectable via distal cues (i.e., odors, sounds, sight) before they contact the body to activate proximal senses (taste, touch), and associative learning is sensitive to this temporal order (Timberlake, 1994). According to the argument being raised here, associative learning will occur selectively for distal signals that provide anticipatory cues to biologically important stimuli, and habituation will follow otherwise. Finally, it should be noted that by the very definition of Pavlovian conditioning, this argument applies to distal stimuli that are neutral prior to conditioning, i.e., stimuli that do not by themselves evoke an appetitive or aversive response, but acquire the ability to do so after being paired with a US.

## Modulation of distal stimulus processing by proximal inputs as a general principle for the organization of learning and behavior

All behaviors can be coarsely classified into approach and avoidance where the animals choose to respond in a manner to maintain or terminate the impact of impinging stimuli. As classical ethologists of the last century astutely documented (Tinbergen, 1951), approach-based behavior systems (e.g., foraging and mating where the animals' movements are directed toward appetitive stimuli, and aggression where the animal approaches aversive stimuli) have two properties in common: The first is the sequential pattern of behavior which requires persistent sign tracking and stimulus evaluation, presenting the animal with multiple check points before it finally commits the consumatory act. The second is both state- and stimulus-dependence of performance, which attunes behavioral choice to physiological demands.

Often referred to as motivation, the impact of internal state variables on behavior is robust to fluctuations in environmental inputs, maintaining the coherence and goal-directedness of behavior in the face of environmental perturbations. Interestingly, when animals are in a conflict between acting in accordance with the existing motivational state or responding to an incompatible external stimulus, their choice reflects a high impact of internal state variables. Similarly, proximal stimuli are more likely to control behavior when they are in conflict with distal stimuli. For example, initiation of swimming in response to noxious mechanical stimulation is suppressed in hungry, but not sated leech (Gaudry and Kristan, 2012). Similarly, male fruit flies fail to escape from mechanical turbulence or dangerously high ambient temperatures when they are mating (Crickmore and Vosshall, 2013). Other examples abound in nature: The type and intensity of the behavioral response to a conspecific is highly modulated both by the motivational state of the animal, and the distance of the intruder. These observations suggest a body-centered hierarchy of impact in the decreasing order of internal state, proximal, and distal stimuli, where closeness to the body yields not only a higher probability of access to behavioral control, but also the potential to modulate the effects of more distal input sources.

One would only expect that the organization of learning mirrors the organization of behavior. Experience dependent change in the feeding reflexes of fruit flies provides a good example. Flies respond to appetitive stimulation of their gustatory receptors by extending their mouthparts, the proboscis. In the presence of a tastant, elicitation of the proboscis extension reflex (PER) is a probabilistic process that is modulated by stimulus properties (e.g., type and concentration of the appetitive solution Dahanukar et al., 2007), physiological state (e.g., hunger Inagaki et al., 2012; Marella et al., 2012, nutrition Edgecomb et al., 1987, and arousal Dethier, 1974; Vargo and Hirsch, 1982), and memory (Chabaud et al., 2006). For example, habituation of PER is observed upon repetitive stimulation of the tarsal receptors with sucrose in the fruit fly *Drosophila melanogaster*. When flies are re-habituated an hour later, PER probability decreases faster relative to the *de novo* control group that is equated for the level of initial sucrose responsiveness, indicating that habituation memory for PER may last for at least an hour (Cevik and Erden, 2012).

Markedly, expression of both short-term habituation and 1-h habituation memory are strictly dependent on the physiological state of the flies. In the above study (Cevik and Erden, 2012), individual flies exhibited one of three distinct response patterns under a PER habituation protocol following 1–4 h of food deprivation: Some flies were completely non-responsive to sucrose irrespective of its concentration. Another group of flies exhibited the converse response pattern and failed to habituate, and again, their responsiveness did not change with sucrose concentration. Finally, a third group of flies responded to sucrose, and habituated upon repeated stimulation.

Two points are worthy of special emphasis regarding the above data set on PER habituation: First and foremost, the effects of stimulus- and state-dependent factors on sucrose responsiveness are clearly non-additive. That is, stimulus factors are not equivalent with respect to their impact on controlling PER or its experience-dependent modulation when compared to the internal state. In fact, the physiological state determined whether and how the flies would respond to appetitive stimuli: Following 1 h of food deprivation, 60% of flies were completely non-responsive to 600 mM sucrose, as if it was not there. At 4 h of food deprivation, less than 20% of the flies remained non-responsive, whereas 40% of the flies failed to show habituation, and continued to respond as if there was nothing else but sucrose. Clearly, state-dependence of responsiveness had a qualitative, but not quantitative effect on how appetitive stimuli were processed for immediate responding as well as for memory-formation.

Second, habituation, when it was observed, was rapid. It can be argued that stimuli exert the highest influence on behavior when they are novel. If a fly emitted at least one PER during the habituation session, its first response was highly likely to occur within the first two trials. To put it in other words, if a fly did not respond in the first few instances of sucrose presentation, it was not likely to respond afterwards. Because the flies are not permitted to ingest sucrose or even touch the sucrose solution with their proboscis in a habituation session, PER emissions do not result in feeding. Under these conditions, tarsal stimulation with sucrose is redundant, and entails rapid habituation. Notice that although neural adaptation and habituation are not dissociable, habituation cannot be reduced to adaptation either. For example, in the above experiment, the same stimulus repetition protocol which supposedly produced similar rates of input adaptation, led to different rates of habituation as a result of hunger modulation. Further, both habituation and its failure could be predicted in advance by early response parameters that were not confounded by adaptation. For example, responsiveness within the first two trials could predict the subsequent pattern of habituation before adaptation was in effect. In general, although short-term habituation is correlated with failures of sensory transmission (Malkinson and Spira, 2013), longer term habituation cannot be reduced to either receptor adaptation or a depression of the sensorimotor synapses, presenting *prima facie* evidence for experience dependent change in the likelihood of responding to the same stimulus (Glanzman, 2009).

In fact, habituation might be the default fate for most stimuli in the absence of top-down modulation (although see Horn and Hinde, 1970 for examples of non-habituating reflexes). To state it in more general terms, habituation is not just a waning of responsiveness for repetitive external stimuli, but is a default property of central processing unless it is modulated by a sensitizing input. This framework might be useful in understanding organization of behavior and learning. Below, I present a brief review of recent findings on appetitive olfactory conditioning in fruit flies to suggest that classical conditioning ensues when the habituation of a distal stimulus is prevented by virtue of its anticipatory pairing with the proximal, sensitizing stimulus under the permissive context of the internal milieu.

## Appetitive olfactory conditioning as a model to study cross-modal stimulus integration

In an appetitive olfactory conditioning paradigm, a group of flies are first exposed to an odor, CS+, which is simultaneously presented with sucrose, US. In alternating trials, they are also exposed to another odor, CS−, which is not accompanied by an appetitive US. During the test phase, the flies are simultaneously presented with CS+ and CS− in the absence of a US, where they choose to approach CS+ following successful conditioning. Notice that the training protocol equates the two olfactory stimuli, CS+ and CS−, with respect to habituation while differentiating them in terms of the sucrose-driven sensitization that follows (Tully and Quinn, 1985).

Mushroom bodies (MB) are bilateral multi-modal sensory integration sites (Strausfeld et al., 2003) that have been associated with appetitive and aversive memories (Mizunami et al., 1998; Keene and Waddell, 2007; Zhang et al., 2013), attention (van Swinderen, 2007), and context-(Liu et al., 1999) and salience-based decision making (Zhang et al., 2007) in several insect species. The MB in each hemisphere of the adult fruit fly brain house ~2500 Kenyon cells which form lobular structures that can be distinguished in terms of their morphology and function. For example, the axons of a subset of Kenyon cells bifurcate to form the vertical α and medial β lobes, another subset likewise forms the α' and β' lobes, and γ neurons have unbranched axons that project medially. Kenyon cell dendrites are housed in the calyces where they receive olfactory input from the projection neurons that ascend from the antennal lobes. In turn, Kenyon cell output converges on a small number (~30 pairs) of extrinsic neurons that innervate distinct areas along MB lobes (Chen et al., 2012; Pai et al., 2013; Placais et al., 2013). Finally, at both the calycal input and the lobular output areas, Kenyon cells make extensive pre- and post-synaptic contacts with aminergic and peptidergic modulatory neurons that relay the computed impact of proximal (i.e., gustatory and tactile) input as well as the internal state (Krashes et al., 2009; Mao and Davis, 2009; Pitman et al., 2011; Aso et al., 2012; Burke et al., 2012).

The innervation pattern of modulatory neurons shows extensive overlap with those of the extrinsic output neurons, defining lobular areas that are functionally specialized for different types and/or stages of memory processing (Krashes et al., 2007; Aso et al., 2012; Xie et al., 2013). For example, a broad, non-selective activation of the α'β' lobes is observed immediately following the training phase of olfactory conditioning. This activity overlaps in space and time with the activity of octopaminergic (OA) neurons that are incited by the sweet taste of the sugar (US), which can modulate conditioned odor preference for as short as a few minute. Longer lasting memories require the activity of dopaminergic (DA) neurons in the PAM cluster (Burke et al., 2012; Liu et al., 2012). A subset of PAM-DA neurons receive input from both the OA neurons that relay the impact of sweetness, and a yet unidentified source that relays information about the nutritive quality of sugar shortly after ingestion. Being the convergence point of the sensory intensity and nutritive quality of food, DA neurons of the PAM cluster in turn innervate the tip of the β' and γ lobes to support olfactory memories that may last for hours (Burke et al., 2012).

The sequential pattern of OA and DA modulation of the Kenyon cells which is required for the formation of appetitive memories can be interpreted as a selection-for-not-habituation of olfactory representations as they are modulated by sensitizing input from progressively more proximal sources. For example, sweetness is a proximal (yet external) input that provides information about the concentration of food, and it can facilitate the associability of mushroom body olfactory representations for minutes via OA modulation. More stable memories require sensitizing modulation from an internal source: DA neurons that relay the impact of the nutritive quality of food can support olfactory memories for hours. Hence, following conditioning, when animals encounter distal olfactory stimuli, they learn to anticipate the sequential arrival of progressively more proximal inputs with positive valence, namely sweetness and nutritive value, through the DA-mediated olfactory associations (see Wittmann et al., 2005; Tully et al., 2007; Howe et al., 2013 for similar examples in the vertebrate brain). Converging evidence comes from a study which showed that in addition to their well-known roles in associative learning, MBs are also involved in sustaining the impact of biologically important stimuli. The disruption of MB function results in a premature habituation to electric shocks that can otherwise function as US's to support aversive olfactory associations (Acevedo et al., 2007).

A similar argument can hold for the retrieval of appetitive olfactory memories. Memory retrieval is inferred as the animal performs the CR; so by definition, it is a process that guides action selection. Therefore, one can imagine that upon encountering the CS, the process of memory retrieval initiates dynamics that bias responding in favor of US anticipation or consummation. In the fly brain, the αβ lobes have been shown to be required for the retrieval of short- and long-term memories (Aso et al., 2012; Xie et al., 2013), so arguably, these lobes are involved in accessing associations of olfactory stimuli to guide anticipatory action selection, irrespective of the temporal phase or stability of memory traces. In accordance with the model being proposed here, it is only expected then, that the αβ lobes would be modulated by the impact of proximal and internal inputs. Interestingly, a zone located at the heel of MB's where α and β lobes intersect, acts as a switch that biases memory-driven performance in favor of approach or avoidance responses. This zone is innervated by a subset of DA neurons of the PPL1 cluster, MB-MP1 neurons, activation of which is necessary for the retrieval of aversive olfactory memories, i.e., for the selection of actions that are compatible with moving away from the odor source (Mao and Davis, 2009; Xie et al., 2013). However, these MB-MP1 neurons can be inhibited by inputs that convey the impact of proximal appetitive substances as well as the internal milieu that bias action selection in favor of approach. For example, information about the sweetness of the tastant (via the OA neurons Burke et al., 2012) and the physiological state of the fly (via neuropeptide F Krashes et al., 2009) converge to inhibit the activity of a subset of PPL1-DA neurons that convey negative valence. This pattern of connectivity reduces the probability of moving away from high-quality tastants and increases the probability of approaching a food source using learned associations, when the animal is hungry.

Notice that the hierarchical modulation of olfactory representations in downstream multi-modal association areas confers flexibility and context-dependence to conditioned responding (Strausfeld, 2012). Being driven both by the internal and proximal environment of the animal, DA neurons modulate the processing of distal stimuli to confer context-dependent salience to a selected subset (i.e., attention), endure the impact of previously inconspicuous distal stimuli by associating them with significant proximal events (i.e., conditioning and memory formation), and mediate the selection of a general action plan *vis a vis* distal stimuli (i.e., approach vs. avoidance).

Finally, it should be noted that the current model suggests a resemblance between the properties of neural circuits that underlie associative learning in vertebrate and invertebrate brains. NMDA-receptor mediated long term potentiation (LTP) has been the principal model of memory formation in the mammalian brain since its discovery by Bliss and Lomo (1973). In the excitatory synapses that undergo LTP, a weak input can become potentiated as a consequence of being coincident with a stronger input (e.g., Barrionuevo and Brown, 1983); and this associative property has validated LTP as a cellular model of Pavlovian conditioning. Although the role of monoamines in environment-specific drug effects and reinforcement learning have long been established as models of conditioned responding in the mammalian brain (e.g., Stewart and Vezina, 1988), monoaminergic modulation of LTP induction has caught researchers' interest more recently. For example, monoaminergic enhancement of hippocampus-dependent memory formation is observed when the emotional valence of the US is relevant (Wittmann et al., 2005), and monoamine transmission is often heterosynaptically activated by the basolateral nucleus of the amygdala (BLA) (Tully et al., 2007). Interestingly, bilateral lesions of BLA produce symptoms that resemble the Kluver-Bucey syndrome (Davis and Whalen, 2001). This condition was originally described when the bilateral removal of temporal lobes including the amygdala caused monkeys to compulsively attend to almost every visual stimulus and proceed to examine even repulsive objects by mouth, increase heterosexual and homosexual behavior, and approach conspecifics as well as human caretakers with a marked absence of anger or fear. In essence, these symptoms reveal a failure to habituate to distal inputs, which results in an inappropriate progression toward initiating and maintaining proximal contact with stimuli irrespective of their incentive value. Similarly, bilateral lesions of BLA (Hatfield et al., 1992), or the depletion of DA and NE in the amygdala (Fernandez-Ruiz et al., 1993) produces a decrement in taste-potentiated odor aversion, but not in taste-aversion *per se*. These results suggest that the monoamine modulation in the BLA functions to guide conditioned avoidance reactions toward distal (e.g., olfactory) stimuli by the signaling the valence of proximal inputs (e.g., taste). Interestingly, olfactory conditioning in fruit flies has recently been shown to involve post-synaptic plasticity in the dendrites of MB output neurons that express NMDA receptors (Xia et al., 2005; Chen et al., 2012; Pai et al., 2013), which closely overlap with the arborizations of DA neurons along the lobes. It can then be argued, that input from proximal receptors initiates monoamine signaling, which in turn modulates excitatory synapses to establish conditioned approach or avoidance of distal stimuli in both vertebrate and invertebrate brains.

## Summary and conclusion

In this brief review, I argued that the impact of a stimulus on behavior and its potential to modulate the effects of other stimuli increase as its distance from the body decreases, and the body-centered hierarchy of stimulus impact applies to the organization of behavior as well as its experience dependent change. For example, the likelihood of habituation, i.e., the inability of a perceivable stimulus to control behavior is higher for distal stimuli, whereas the likelihood of sensitization is higher for stimuli that come into direct contact with the body to activate gustatory and/or mechanoreceptors. Further, I argued that mechanisms of associative learning are not independent from those of habituation and sensitization. Pavlovian conditioning ensues when internal state (e.g., hunger) up-regulates the sensitizing potential of the proximal US (e.g., food), which in turn prevents habituation to the distal CS (e.g., odor). According to the argument being raised here, CS−US pairings should fail to yield associative learning if sensitization does not occur to the US. In this short review, I gave examples from olfactory learning in fruit flies, but I believe that a similar hierarchy of body-centered stimulus impact exists in the vertebrate brain as well. The idea that sensitization from proximal inputs and the internal milieu drives associative learning might help our understanding of phenomena such as the development of chronic pain (Agroff et al., 2009), or eating disorders (Kaye et al., 2011) as failures of habituation.

### Conflict of interest statement

The author declares that the research was conducted in the absence of any commercial or financial relationships that could be construed as a potential conflict of interest.
